# Clinical Effects of Stereotactic Body Radiation Therapy Targeting the Primary Tumor of Liver-Only Oligometastatic Pancreatic Cancer

**DOI:** 10.3389/fonc.2021.659987

**Published:** 2021-05-27

**Authors:** Xiaoqin Ji, Yulu Zhao, Chenglong He, Siqi Han, Xixu Zhu, Zetian Shen, Cheng Chen, Xiaoyuan Chu

**Affiliations:** ^1^ Department of Radiation Oncology, Jinling Hospital, Nanjing Clinical School of Nanjing Medical University, Nanjing, China; ^2^ Department of Medical Oncology, Jinling Hospital, Nanjing Clinical School of Nanjing Medical University, Nanjing, China; ^3^ Department of Medical Oncology, Jinling Hospital, First School of Clinical Medicine, Southern Medical University, Nanjing, China

**Keywords:** pancreatic cancer, metastasis, stereotactic body radiotherapy, CyberKnife, pain

## Abstract

**Aim:**

To investigate the efficacy and safety of stereotactic body radiotherapy (SBRT) targeting the primary tumor for liver-only oligometastatic pancreatic cancer.

**Methods:**

We compared the efficacy and safety of SBRT plus chemotherapy with chemotherapy alone in patients with liver-only oligometastatic pancreatic cancer. The populations were balanced by propensity score-weighted and propensity score-matched analyses based on baseline variables. The primary outcome was overall survival (OS). The secondary outcomes included progression free survival (PFS), local progression, metastatic progression and symptomatic local control.

**Results:**

This is a retrospective study of 89 pancreatic cancer patients with liver-only oligometastasis. Overall, 34 (38.2%) and 55 (61.8%) patients received SBRT plus chemotherapy and chemotherapy alone, respectively. After propensity score matching, 1-year OS rate was 34.0% (95%CI, 17.8-65.1%) in the SBRT plus chemotherapy group and 16.5% (95%CI, 5.9-46.1%) in chemotherapy alone group (P=0.115). The 6-month PFS rate was 29.4% (95%CI, 15.4-56.1) in SBRT plus chemotherapy and 20.6% (95%CI, 8.8-48.6) in chemotherapy alone group (P=0.468), respectively. Further subgroup analysis indicated that the addition of SBRT improved OS in patients with primary tumor located in the head of pancreas (stratified HR, 0.28; 95% CI, 0.09 to 0.90) or good performance status (stratified HR, 0.24; 95% CI, 0.07 to 0.86). In terms of disease control, SBRT delayed local progression of pancreas (P=0.008), but not distant metastatic progression (P=0.56). Besides, SBRT offered significant abdominal/back pain relief (P=0.016) with acceptable toxicities.

**Conclusions:**

The addition of SBRT to chemotherapy in patients with liver-only oligometastatic pancreatic cancer improves the OS of those with primary tumor located in the head of pancreas or good performance status. In addition, it is a safe and effective method for local progression control and local symptomatic palliation in patients with metastatic pancreatic cancer.

## Introduction

Pancreatic cancer has an extremely poor prognosis with a 5-year survival rate of 9% (1). Since the disease presents few, if any, symptoms before it progresses to advanced stage, approximately 80-85% of patients present with Stage III or IV disease at the time of initial diagnosis (2, 3). For metastatic pancreatic patients, the 5-year overall survival (OS) rate is extremely low (about 3%) (1). Systemic chemotherapy combinations, including FOLFIRINOX (5-fluorouracil, folinic acid, irinotecan, and oxaliplatin) and gemcitabine plus nab-paclitaxel (GT), have emerged as standards of care of front-line therapy, increased survival with a median of 11.1 and 8.5 months, respectively (4, 5). In addition, the exploration of immunotherapy and targeted therapy has provided new treatments for these patients (6–9).

The innervation of pancreatic tissue composes the network of sympathetic and parasympathetic systems, yielding an increase in pain sensitivity (10). In most patients with pancreatic cancer, local tumor progression often cause severe symptoms, including abdominal and back pain, biliary obstruction, and pancreatic insufficiency, which severely affect patients’ quality of life (2, 6). Some studies indicate that radiation therapy has shown efficacy in improving local control, delaying disease progression and ameliorating local symptoms for pancreatic cancer (11, 12).

Additionally, the locally destructive growth of primary tumor was a significant cause of death for many patients with pancreatic cancer (13). Therefore, local treatment may reduce primary tumor burden and provide better disease control, thereby improving clinical outcomes. You et al. reported that locoregional radiotherapy added to chemotherapy significantly improves OS in chemotherapy-sensitive patients with metastatic nasopharyngeal carcinoma (14). Rusthoven et al. utilized the National Cancer Database (NCDB) and found that compared with androgen deprivation alone, the addition of prostate radiotherapy substantially prolonged the OS of men with metastatic prostate cancer (15). Parker et al. reported that radiotherapy to the primary tumor did not improve OS in patients with newly diagnosed metastatic prostate cancer, but improved failure-free survival (16). Local failure is not a common cause of death in prostate malignancy. However, local progression in pancreatic cancer patients may have significant morbidity and mortality. Thus, in patients with mestastatic pancreatic cancer, radiation therapy targeting the primary tumor may have a different rationale. In the last few years, stereotactic body radiotherapy (SBRT) has emerged as a local treatment for pancreatic cancer with local control rate exceeding 90% at 1 year (17, 18). However, there is few research on the application of SBRT to the primary tumor for metastatic pancreatic cancer.

In this study, we retrospectively investigated the efficacy and safety of SBRT to primary tumor with chemotherapy *vs* chemotherapy alone in patients with liver-only oligometastatic pancreatic cancer at initial diagnosis.

## Methods

### Patients

This retrospective study was conducted on 89 pancreatic cancer patients with liver-only metastasis at initial diagnosis from January 2010 to December 2019 in Jinling Hospital. They were treated with systemic chemotherapy alone or plus SBRT delivered to the primary tumor. The inclusion criteria of the patients were: (1) Histologically or cytologically confirmed, or clinically diagnosed according to our clinical diagnosis criteria, including typical pancreatic cancer symptoms (abdominal/back pain) and positive carbohydrate antigen 19–9 (CA19-9) value, computed tomography (CT), magnetic resonance imaging (MRI) and 18-fluorodeoxyglucose positron emission tomography/computed tomography (^18^F-FDG-PET/CT); (2) Oligometastases, with a maximum of 5 metastases in the liver (< 4 cm in size); (3) Comprehensive clinical and imaging examinations prior to treatment proved to be accompanied by liver metastasis at initial diagnosis; (4) Patients who had previously been treated with abdominal radiotherapy, and had a synchronous abdominal cancer or other cancers requiring treatment were excluded. The study was approved by the Ethics Committee of our institution, and written informed consents were obtained from all patients. The main characteristics of all patients are summarized in **Table 1**. Before treatment, data were collected, such as performance status of Eastern Cooperative Oncology Group (ECOG), age, baseline serum carbohydrate antigen 19–9 (CA19-9) concentration, T and N stages.

**Table 1:** Comparison of baseline variables between SBRT plus chemotherapy and chemotherapy alone groups in the original and matched data sets.

	Unmatched cohort			Propensity-score-matched cohort		
Characteristic	SBRT plus chemotherapy (n = 34)	Chemotherapy alone (n = 55)	P	SBRT plus chemotherapy (n = 23)	Chemotherapy alone (n = 23)	P
Age (years)			0.802			0.768
≤60	17 (50%)	26 (47.3%)		11 (47.8%)	12 (52.2%)	
>60	17 (50%)	29 (52.7%)		12 (52.2%)	11 (47.8%)	
Gender			0.657			0.345
Male	22 (64.7%)	33 (60.0%)		14 (60.9%)	17 (73.9%)	
Female	12 (35.3%)	22 (40.0%)		9 (39.1%)	6 (26.1%)	
Year of diagnosis			0.007			0.760
2010-2014	21 (61.8%)	18 (32.7%)		15 (65.2%)	14 (60.9%)	
2015-2019	13 (38.2%)	37 (67.3%)		8 (34.8%)	9 (39.1%)	
Diagnostic mode			0.514			—
Clinical	5 (14.7%)	7 (12.7%)		0 (0%)	0 (0%)	
Histological/cytological	29 (85.3%)	48 (87.3%)		23 (100%)	23 (100%)	
Performance status			0.030			1.000
0-1	13 (38.2%)	34 (61.8%)		9 (39.1%)	9 (39.1%)	
2	21 (61.8%)	21 (38.2%)		14 (60.9%)	14 (60.9%)	
Primary pancreatic tumor location			0.059			1.000
Head	18 (52.9%)	18 (32.7%)		10 (43.5%)	10 (43.5%)	
Body/tail	16 (47.1%)	37 (67.3%)		13 (56.5%)	13 (56.5%)	
Pre-treatment CA19–9 (U/ml)			0.103			1.000
≤1000	19 (55.9%)	21 (38.2%)		12 (52.2%)	12 (52.2%)	
>1000	15 (44.1%)	34 (61.8%)		11 (47.8%)	11 (47.8%)	
T category^※^			0.030			0.765
T3	17 (50.0%)	40 (72.7%)		14 (60.9%)	13 (56.5%)	
T4	17 (50.0%)	15 (27.3%)		9 (39.1%)	10 (43.5%)	
N category^※^			0.676			0.767
N0	17 (50.0%)	25 (45.5%)		11 (47.8%)	10 (43.5%)	
N1	17 (50.0%)	30 (54.5%)		12 (52.2%)	13 (56.5%)	
Chemotherapy regimen			0.198			1.000
GT	2 (5.9%)	1 (1.8%)		2 (8.7%)	1 (4.3%)	
Gemox	11 (32.4%)	13 (23.6%)		6 (26.1%)	6 (26.1%)	
GS	9 (26.5%)	25 (45.5%)		7 (30.4%)	8 (34.8%)	
GP	3 (8.8%)	1 (1.8%)		2 (8.7%)	1 (4.3%)	
G	9 (26.5%)	13 (23.6%)		6 (26.1%)	7 (30.4%)	
Others	0 (0.0%)	2 (3.6%)		0 (0.0%)	0 (0.0%)	
Chemotherapy cycles			0.777			0.873
1	4 (11.8%)	5 (9.1%)		3 (13.0%)	5 (21.7%)	
2	11 (32.4%)	13 (23.6%)		8 (34.8%)	5 (21.7%)	
3	5 (14.7%)	7 (12.7%)		3 (13.0%)	3 (13.0%)	
4	8 (23.5 %)	15 (27.3%)		6 (26.1%)	6 (26.1%)	
>4	6 (17.6%)	15 (27.3%)		3 (13.1%)	4 (17.4%)	

CA19-9, carbohydrate antigen 19–9; SBRT, stereotactic body radiotherapy; GT, gemcitabine and nab-paclitaxel; Gemox, gemcitabine plus oxaliplatin; GS, gemcitabine and S-1; GP, gemcitabine and nedaplatin; G, gemcitabine. ^※^According to the American Joint Committee on Cancer and the Union for International Cancer Control stage system (7th edition).

### SBRT

The study used CyberKnife (Accuray Incorporated, Sunnyvale, CA, USA) for localized treatment. Firstly, all patients were implanted with 1-3 gold markers (5.0 × 0.8 mm) under ultrasound or CT guidance. The gold fiducials were placed in the lesion. Then, when the gold fiducial was firmly attached to the surrounding tissue (approximately 7 days), abdominal CT scan (Brilliace Big Bore 16CT Philips Germany) was performed. Before CT positioning, patient was fasted for more than 4 hours. 100-150 ml of oral contrast agent was taken 30, 20 and 10 minutes before the CT scan to clearly show the gastrointestinal tract. Besides, intravenous contrast was also used to better display the lesions. The CT scan range is 15 cm above and below the pancreatic lesion, and the layer thickness is 1 mm.

The gross tumor volume (GTV) was the primary tumor of the pancreas and enlarged lymph nodes (defined as short axis diameter ≥ 1 cm, or PET positive) observed through the imaging. MRI or 18F-FDG-PET/CT were used for target delineation. Radiation oncologists delineated GTV on axial slices of the contrast-enhanced CT. Since our center performs tracking (Synchrony), internal target volume (ITV) is not requried. The clinical tumor volume (CTV) was equivalent to GTV. The planning tumor volume (PTV) margin was 0-5 mm from the GTV, depending on the disease location and size. We used oral meglumine diatrizoate to clearly display the gastrointestinal tract and MRI images to determine the junction between tumor and gastrointestinal structures, thereby helping to modify PTV to avoid overlapping of gastrointestinal organs. Average total prescribed dose was 41.1 gray (Gy) (range of 25-50 Gy), which was given in 5-7 fractions. Because the median number of fractions was 5, organs at risk (OAR) dose constraints applied for five fraction SBRT was used in this study. The dose-volume constraints for OARs are summarized in **Appendix Table 1**. Respiration synchronous tracking (Synchrony) was used to track the movement of the fiducials for simultaneous irradiation. The delivery of SBRT was performed between cycles of chemotherapy, usually once a day. SBRT usually takes about 1 h, and it is difficult for patients with severe pain to maintain the same posture over a long time. Thus, 10 mg of morphine were taken half an hour before SBRT to relieve the patient’s pain and help complete the treatment.

### Chemotherapy

Chemotherapy regimens were mostly gemcitabine-based chemotherapy (up to 97.8%), including gemcitabine plus nab-paclitaxel, gemcitabine plus oxaliplatin, gemcitabine plus S-1, gemcitabine monotherapy and so on (**Table 1**). Concurrent administration of systemic therapy and SBRT was avoided if possible. Most patients continue chemotherapy after radiotherapy.

### Symptom Assessment

Before treatment, patients were asked at baseline to identify a ‘‘target symptom’’ (pain), which is their main complaint that they hope to relieve. At each follow-up visit, they were asked to describe the severity of target symptom compared to baseline. The pain was scored using the visual analogue scale, and was classified into the none (score 0), mild (score 1–3), moderate (score 4–6) and severe pain (score 7–10). The symptom score is always collected as part of the clinical visits.

### Outcomes and Follow Up

After completion of treatment, patients were followed-up every 3-5 weeks in the first 6 months and every 3 months afterwards until the death. Treatment results and side effects were evaluated on the basis of clinical examinations, laboratory examination, CT, MRI, bone scan, and ^18^F-FDG-PET/CT. Toxicity was evaluated according to the National Cancer Institute Common Terminology Criteria for Adverse Events version 5.0. The primary efficacy outcome was OS, defined as the time from the start of treatment to the death due to any cause. Secondary outcomes included progression-free survival (PFS) (defined as the time from the start of treatment to progression at any site or death), local progression (defined as the progression of tumors in the pancreas from the start of treatment) and metastatic progression (defined as new metastases or progression of existing metastases from the start of treatment). Death without the event of interest was a competing event, and patients lost to follow-up without the event were censored.

### Statistical Analysis

We compared baseline and matched characteristics using Pearson χ² or Fisher’s exact test for categorical data. To address the imbalance of potential confounders between the SBRT plus chemotherapy and chemotherapy alone groups, propensity scores-matched analysis was performed for treatment groups (19). The propensity score model included T stage, N stage, gender, age, performance status, primary pancreatic tumor location, CA19–9, and year of diagnosis. Then, matched pairs were formed between patients treated by SBRT plus chemotherapy and those treated by chemotherapy alone using a one-to-one nearest neighbor calliper with width of 0.3 (the maximum allowable difference in propensity scores). On the basis of the propensity score matching, a stabilized inverse probability of treatment weighting (IPTW) was calculated (20, 21). Weights were truncated at the 5th and 95th percentile to reduce potential data sparsity. To assess balance before and after matching and weighting, the standardized mean difference (SMD) was calculated. SMD value of 0.1 or less indicated optimal balance. Kaplan-Meier estimators were calculated for each group and were compared using the log-rank test. Cox proportional hazards regression model was used to compare the relative treatment efficacy between treatment groups. Within the matched patient group, the heterogeneity of treatment efficacy was assessed with tests of interaction and subgroup analyses, which explored the effect of gender, age, performance status, primary pancreatic tumor location, CA19–9, year of diagnosis, T stage and N stage. An HR less than 1.00 favored SBRT plus chemotherapy. Competitive risk analysis (Gray’s test) (22) was used to estimate the cumulative incidence of local progression for pancreatic lesions and the cumulative incidence of metastatic progression. Statistical analysis was done using SPSS version 24.0 and R version 3.6.3. All tests were displayed on both sides, with 95% CIs and relevant p values.

## RESULTS

### Patient Characteristics and Treatment Features

Between January 01, 2010, and December 31, 2019, 89 patients with metastatic pancreatic cancer were included in this study, of whom 34 received SBRT plus chemotherapy and 55 received chemotherapy alone. The median time interval from diagnosis to the start of treatment was 8 days (0-28 days). The baseline characteristics of all patients were presented in **Table 1**. Patients who received SBRT plus chemotherapy had a higher ratio of poor performance status (ECOG=2, 61.8% *vs* 38.2%, P=0.030) and advanced T stage (T4, 50.0% *vs* 27.3%, P=0.030). Patients were more likely to receive SBRT from 2010 to 2014 (Year of diagnosis, 61.8% *vs* 32.7%, P=0.007). To eliminate the influence of these differences on subsequent analysis, we matched the two groups for all covariates by propensity score matching (**Appendix Table 2** and **Appendix Figure 1**). The baseline characteristics were well balanced between two groups after matching (**Table 1**).

**Figure 1:**
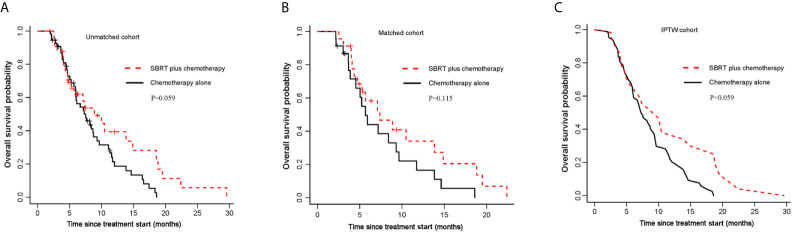
Kaplan-Meier curves for OS. **(A)** OS of the unmatched cohort; **(B)** shows OS of the propensity score matched group; **(C)** shows OS of the inverse probability of treatment weight-adjusted group. SBRT, stereotactic body radiotherapy.

Before SBRT, 12 patients received no chemotherapy, 13 patients received 1-cycle of chemotherapy, 5 patients received 2-cycles chemotherapy, and 4 patients received ≥ 3-cycles chemotherapy. The median number of chemotherapy cycles was 1 (range of 0-6) before SBRT. Almost all patients received systemic chemotherapy after radiotherapy. The median time interval from initial treatment to SBRT was 13 days (0-114 days). The median PTV was 77.5 cm3 (range of 17.8-355.7 cm3). The treatment duration was 5-9 days. Median total prescribed dose was 42.5 gray (Gy) (range of 25-50 Gy), which was given in 5-7 fractions. The median prescription isodose was 73%. The SBRT planning and delivery variables are shown in **Appendix Table 3**.

### Survival Analysis

Median follow-up time for all patients was 20.9 months (95% CI,17.7-24.1 months). In unmatched analysis, the median OS was 8.9 months (95% CI, 5.7-18.8 months) for SBRT plus chemotherapy group and 7.5 months (95% CI, 6.0-9.6 months) for chemotherapy alone group. The 1-year OS rate was 39.4% (95% CI, 24.1–64.3%) for SBRT plus chemotherapy group and 21.3% (95% CI, 11.9–38.0) for chemotherapy alone group. Compared with the control group, the SBRT group has no survival advantage (log-rank P=0.059; **Figure 1A**; **Table 2**). This is consistent with the result of the propensity-score-matched analysis. The rates of OS at 1-year survival for SBRT plus chemotherapy and chemotherapy alone groups were 34.0% (95%CI, 17.8-65.1%) and 16.5% (95%CI, 5.9-46.1%), respectively (log-rank P= 0.115; **Figure 1B**; **Table 2**). In the IPTW analysis, SBRT still was not associated with a significant OS benefit. The 1-year OS rate was 38.0% in SBRT plus chemotherapy group versus 22.2% in the chemotherapy alone group (log-rank P=0.059; **Figure 1C**; **Table 2**).

**Table 2:** Summary of estimated treatment effect for main outcome measures in unmatched, propensity Matched and IPTW groups.

	Unmatched			Propensity Matched			IPTW		
	SBRT plus chemotherapy	Chemotherapy alone	P	SBRT plus chemotherapy	Chemotherapy alone	P	SBRT plus chemotherapy	Chemotherapy alone	P
**OS rate**			0.059			0.115			0.059
6-months	62.0%	60.5%		58.3%	44.0%		66.0%	61.9%	
12-months	39.4%	21.3%		34.0%	16.5%		38.0%	22.2%	
**PFS rate**			0.113			0.468			0.093
6-months	29.3%	19.2%		29.4%	20.6%		28.4%	19.3%	
12-months	8.4%	2.4%		0%	5.2%		6.8%	2.1%	
**Local progression rate**			0.016			0.008	–	–	–
6-months	12.6%	34.6%		8.7%	36.7%		–	–	–
12-months	22.1%	53.2%		14.2%	53.3%		–	–	–
**Metastatic progression rate**			0.086			0.56	–	–	–
6-months	61.6%	78.2%		66.2%	71.8%		–	–	–
12-months	88.1%	95.3%		100%	94.4%		–	–	–
**Local symptomatic palliation rate**			0.015			0.016	–	–	–
3-months	78.8%	52.8%		87.0%	54.5%		–	–	–

IPTW, inverse probability of treatment weight; SBRT, stereotactic body radiotherapy.

**Figure 2:**
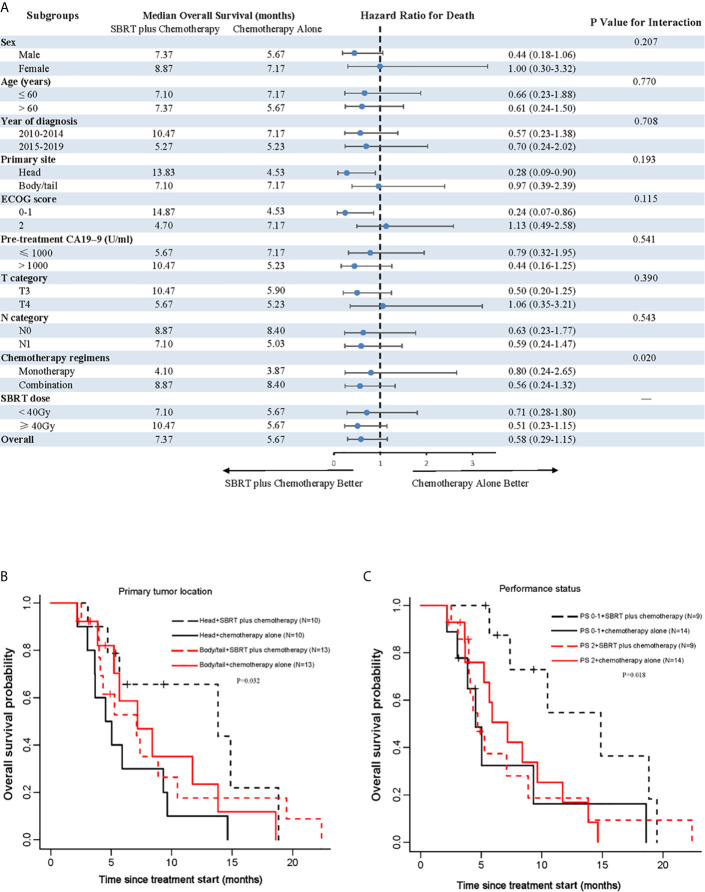
Analyses of OS in matched population. **(A)** Forest plot of subgroup analyses of OS; **(B)** shows OS of the primary tumor location; **(C)** shows OS of performance status. SBRT, stereotactic body radiotherapy; PS, performance status.

To explore whether SBRT would benefit selected patients, we performed subgroup analyses of the matched cohort. The P values for interaction were not significant in most of the prespecified subgroups, indicating that there was no significant difference on OS between subgroups (**Figure 2A**). Notably, the addition of SBRT was beneficial for OS in patients with primary tumor located in the head of pancreas (stratified HR, 0.28; 95% CI, 0.09 to 0.90; P=0.193 for interaction; **Figures 2A**, **B**) or those with good performance status (stratified HR, 0.24; 95% CI, 0.07 to 0.86; P=0.115 for interaction; **Figures 2A**, **C**).

Compared with chemotherapy alone group, the SBRT plus chemotherapy group also did not have the survival advantage on PFS (**Appendix Figure 2**; **Table 2**). Subgroup analyses in the matched cohort showed that there was no beneficial effect of SBRT on PFS across all subgroups (**Appendix** **Figure 3**).

**Figure 3:**
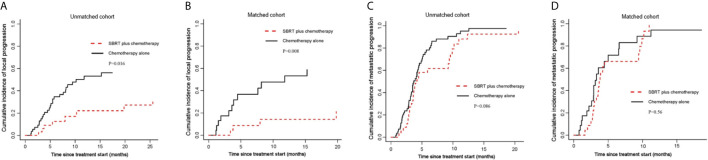
Cumulative incidence curves for the probability of each competing event. **(A)** cumulative incidence of local progression in the unmatched group; **(B)** cumulative incidence of local progression in the matched group; **(C)** cumulative incidence of metastatic progression in the unmatched group; **(D)** cumulative incidence of metastatic progression in the matched group.

### Local Progression and Metastatic Progression

By competing risk analysis in the unmatched groups, the cumulative incidence of local progression within the pancreas was 22.1% (95%CI, 8.2-40.2) for SBRT plus chemotherapy group and 53.2% (95%CI, 37.5-66.6) for chemotherapy alone group at 12 months. The addition of SBRT significantly delayed disease progression in the pancreas (SHR, 0.40; 95% CI, 0.19-0.84; P=0.016; **Figure 3A** and **Table 2**). This is consistent with the result of the propensity-score-matched analysis. The cumulative incidence of local progression in the pancreas was 14.2% (95%CI, 3.2-33.2) for SBRT plus chemotherapy group and 53.3% (95%CI, 27.8-73.4) for chemotherapy alone group at 12 months (SHR, 0.23; 95% CI, 0.08-0.69; P=0.008; **Figure 3B** and **Table 2**).

As for metastatic progression, the cumulative incidence was 61.6% (95%CI, 41.9-76.4) for SBRT plus chemotherapy group and 78.2% (95%CI, 63.8-87.5) for chemotherapy alone group at 6 months in the unmatched groups. The addition of SBRT did not delay metastatic progression (P=0.086; **Figure 3C** and **Table 2**). This is consistent with the result of the propensity-score-matched analysis. The cumulative incidence of metastatic progression was 66.2% (95%CI, 41.6-82.4) for SBRT plus chemotherapy group and 71.8% (95%CI, 44.2-87.4) for chemotherapy alone group at 6 months (SHR, 0.83; 95% CI, 0.44-1.55; P=0.56; **Figure 3D** and **Table 2**).

### Symptom Palliation

**Figure 4:**
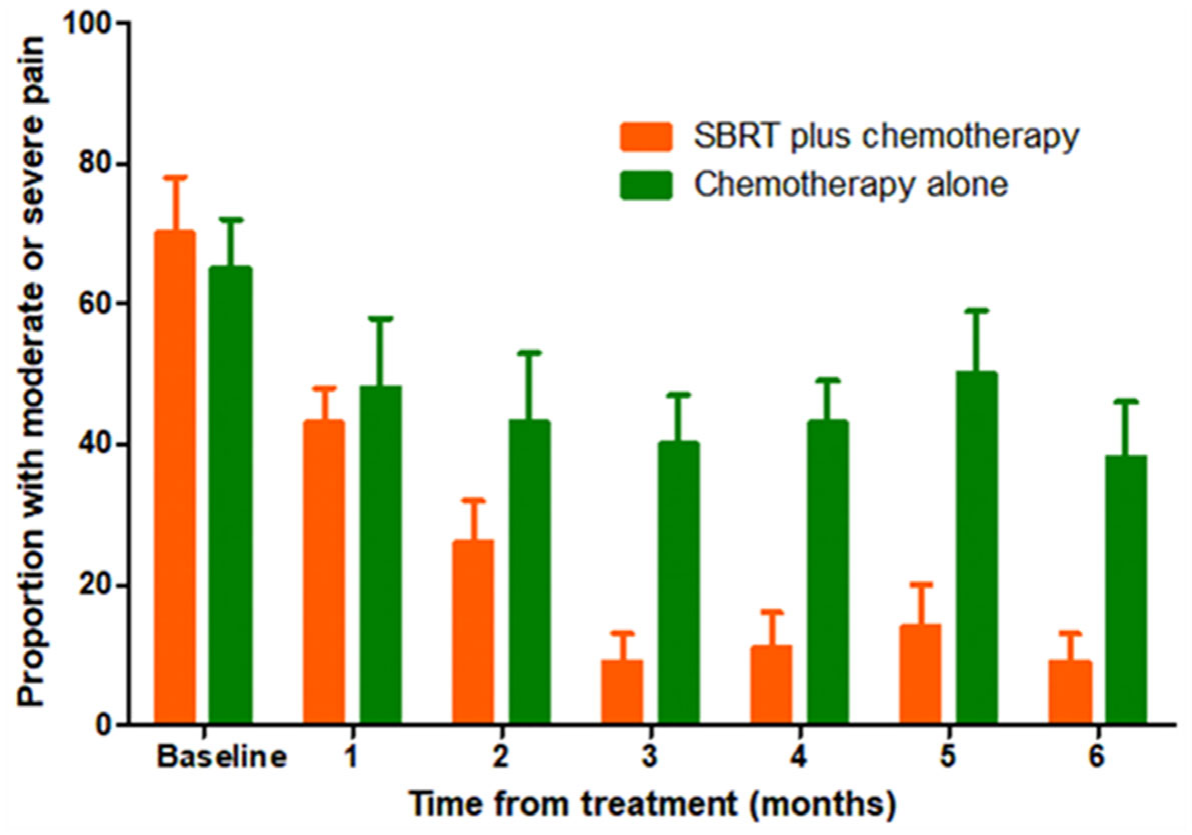
Proportion of patients with moderate or severe abdominal/back pain over time in the matched group.

The definition of symptom palliation is that moderate or severe symptoms at baseline should be improved, mild symptoms should be controlled, and the occurrence of other symptoms should be prevented (23). With these criteria, we compared changes of the pain symptom from baseline to 3 months (improved: moderate or severe at baseline, mild or nil at 3 months; controlled: mild at baseline, mild or nil at 3 months; prevented: nil at baseline, nil at 3 months). Patients who had died by 3 months were considered as without symptom palliation. Symptomatic palliation was assessed using a scoring system, such as visual analogue scoring for pain. After propensity matching, in SBRT plus chemotherapy group, the symptom of 13 patients was improved, that of 7 patients was controlled and that of 0 patient was prevented. In chemotherapy alone group, the symptom of 5 patients was improved, that of 7 patients was controlled, that of 0 patient was prevented, and 1 patient was lost to follow-up. The pain palliation rate was 87.0% for the SBRT plus chemotherapy group and 54.5% for the chemotherapy alone group at 3 months (**Table 2**). Palliation were observed with the addition of SBRT for abdominal/back pain (P=0.016). As shown in **Figure 4**, the proportion of patients with moderate or severe symptoms significantly decreased in SBRT plus chemotherapy group over time.

### Toxicity

Mild toxic effects were recorded for patients, including grade 1 and grade 2 of transient fatigue, anorexia, nausea, and vomiting. Overall, there were no significant differences in hepatotoxic, nephrotoxic, and hematologic toxic effects between two groups. Due to the adverse effects of radiotherapy, one patient presented with duodenal ulcer bleeding (grade 3), and the symptom was improved after endoscopic intervention. Since this patient had a positive history of duodenal ulcer, we suppose that SBRT may cause its recurrence. Thus, for patients with a history of gastric or duodenal ulcers, dose constraints may have to be individualized. The details on the comparison of toxicity between the SBRT plus chemotherapy group and the chemotherapy alone group were summarized in **Appendix Table 4**.

## Discussion

Although chemotherapy remains the primary treatment method for metastatic pancreatic cancer, the use of SBRT has been increasing. However, the clinical efficacy of SBRT to primary tumor for patients with metastatic pancreatic cancer was unclear. To solve this problem, we performed propensity-matched analyses of 89 patients who were newly diagnosed with metastatic pancreatic cancer. These patients were divided into two groups: SBRT plus chemotherapy group and chemotherapy alone group. Our study showed that the addition of SBRT did not improve OS. However, subgroup analysis showed that SBRT improved OS in patients with primary tumor located in the head of pancreas or good performance status. This is probably due to that patients with good fitness can withstand intensive combination therapy. Therefore, consideration of the tumor location and performance status may be a reasonable step towards individualized therapy.

There have been few studies investigating the role of SBRT in the local control of primary tumors of metastatic pancreatic cancer. Lischalk et al. (24) analyzed 20 patients with pathologically diagnosed metastatic adenocarcinoma of the pancreas. SBRT was conducted on the primary pancreatic tumor in five fractions to a total dose of 25-30 Gy. The 1-year local control rate and OS rate were 43% and 53%, respectively. Koong et al. (25) retrospectively analyzed patients with metastatic pancreatic cancer who received stereotactic ablative radiotherapy to the primary tumor. They found the median OS was 7 months, with a cumulative incidence of local failure at 1 year of 25%. However, these studies only had SBRT treatment group and lacked a control group. In our study, patients were divided into SBRT plus chemotherapy group and chemotherapy alone group. The result revealed that the addition of SBRT improved local disease control. However, the improvement in disease control did not transform into a benefit in OS, which may be partly due to the high proportion of patients who developed distant metastatic progression (66.2% *vs* 71.8%, P=0.56).

SBRT to the primary tumor in the case of metastatic pancreatic tumor has a different rationale than the oligometastatic pathway being explored in other disease sites. Many patients with pancreatic malignancy may experience significant morbidity/mortality from local progression of their disease. SBRT in this setting may provide significant benefit. This is different rationale to potentially pursue SBRT than the oligometastatic disease paradigm (SABR-COMET et) that local ablation to all sites may improve outcomes (26, 27). As has been observed recently, radiating a single site in oligometastatic disease is unlikely to provide benefit in patients (28). However, in the context of that single site being a significant cause of morbidity and mortality with local progression, SBRT may provide a significant benefit in this population—but should be tested in a prospective trial.

In addition to the limited life expectancy, the primary pancreatic tumor may cause severe local symptoms, leading to poor life quality (29). Amelioration of symptoms, especially abdominal or back pain, should be given priority in the treatment for metastatic pancreatic cancer. A recent systematic review (10) of the effects of SBRT on pain relief in patients with locally advanced pancreatic carcinoma reported a global overall response rate of 84.9%. Similarly, Su et al. (30) showed that SBRT effectively relieved the abdominal pain of 65% of patients with acceptable toxicities. In our study, in addition to providing good local disease control, SBRT offered improvement in pain control for patients with metastatic pancreatic cancer. Patients with metastatic pancreatic cancer may be treated with SBRT for symptom relief or delaying symptom progression.

This study was mainly limited due to its retrospective nature at a single institution, relatively small sample size, and limited metastatic disease burden with a focus on liver-only oligmetastatic patients. Additionally, there were a variety of types of chemotherapy and number of chemotherapy cycles in this study. However, there was no significant difference in chemotherapy regimens and the number of chemotherapy cycles between two groups. The result in this paper should be further verified with a larger sample size and extended to other metastatic sites of pancreatic cancer.

In conclusion, our study showed a benefit of the combination of SBRT with chemotherapy for pancreatic-specific disease control and palliation of cancer-related symptoms with acceptable toxicities in pancreatic cancer patients with liver-only oligometastasis. Although the addition of SBRT did not improve OS in all patients, it prolonged OS in patients with primary tumor located in the head of pancreas or good performance status. Therefore, the further research is needed to study the role of SBRT in carefully selected patients and as a consolidation therapy after chemotherapy.

## Data Availability Statement

The raw data supporting the conclusions of this article will be made available by the authors, without undue reservation.

## Ethics Statement

The study was approved by the Ethics Committee of Jinling Hospital, and written informed consents were obtained from all patients. The patients/participants provided their written informed consent to participate in this study.

## Author Contributions

XC and CC designed the study. XJ, CH and ZS collected the data. XJ, SH and YZ wrote the manuscript. XC and XZ analyzed and interpreted the data. All authors contributed to the article and approved the submitted version.

## Funding

This work was supported by grants from Natural Science Foundation of Jiangsu Province (BK20181238 to XC).

## Conflict of Interest:

The authors declare that the research was conducted in the absence of any commercial or financial relationships that could be construed as a potential conflict of interest.

## Supplementary Material

Supplementary Figure 1 Plot of the balance evaluated before and after matching/weighting.

Supplementary Figure 2 Kaplan-Meier curves for PFS. **(A)** PFS of the unmatched cohort; **(B)** PFS of the propensity score matched group; **(C)** PFS of the inverse probability of treatment weight-adjusted group. PFS, progression-free survival; SBRT, stereotactic body radiotherapy.

Supplementary Figure 3 Forest plot of PFS subgroup analyses in matched study population. PFS, progression-free survival; SBRT, stereotactic body radiotherapy.

## Abbreviations

SBRT, stereotactic body radiotherapy; PFS, progression-free survival; OS, overall survival; CA19–9, carbohydrate antigen 19–9; CT, computed tomography; MRI, magnetic resonance imaging; ^18^F-FDG-PET/CT, 18-fluorodeoxyglucose positron emission tomography/computed tomography; ECOG, Eastern Cooperative Oncology Group; GTV: gross tumor volume; CTV, clinical tumor volume; PTV, planning tumor volume; BED, biological effective dose; Gy, gray; IPTW, inverse probability of treatment weight; SMD, standardized mean difference. S-1, the prodrug of 5-fluorouracil comprising tegafur, gimeracil, and oteracil; GT, gemcitabine and nab-paclitaxel; GS, gemcitabine and S-1; Gemox, gemcitabine plus oxaliplatin.
