# Supplementary figures and images for: Clinical Effects of Stereotactic Body Radiation Therapy Targeting the Primary Tumor of Liver-Only Oligometastatic Pancreatic Cancer
Source: Front Oncol. 2021 May 27;11:659987. doi: 10.3389/fonc.2021.659987 (PMC8190391; doi:10.3389/fonc.2021.659987)

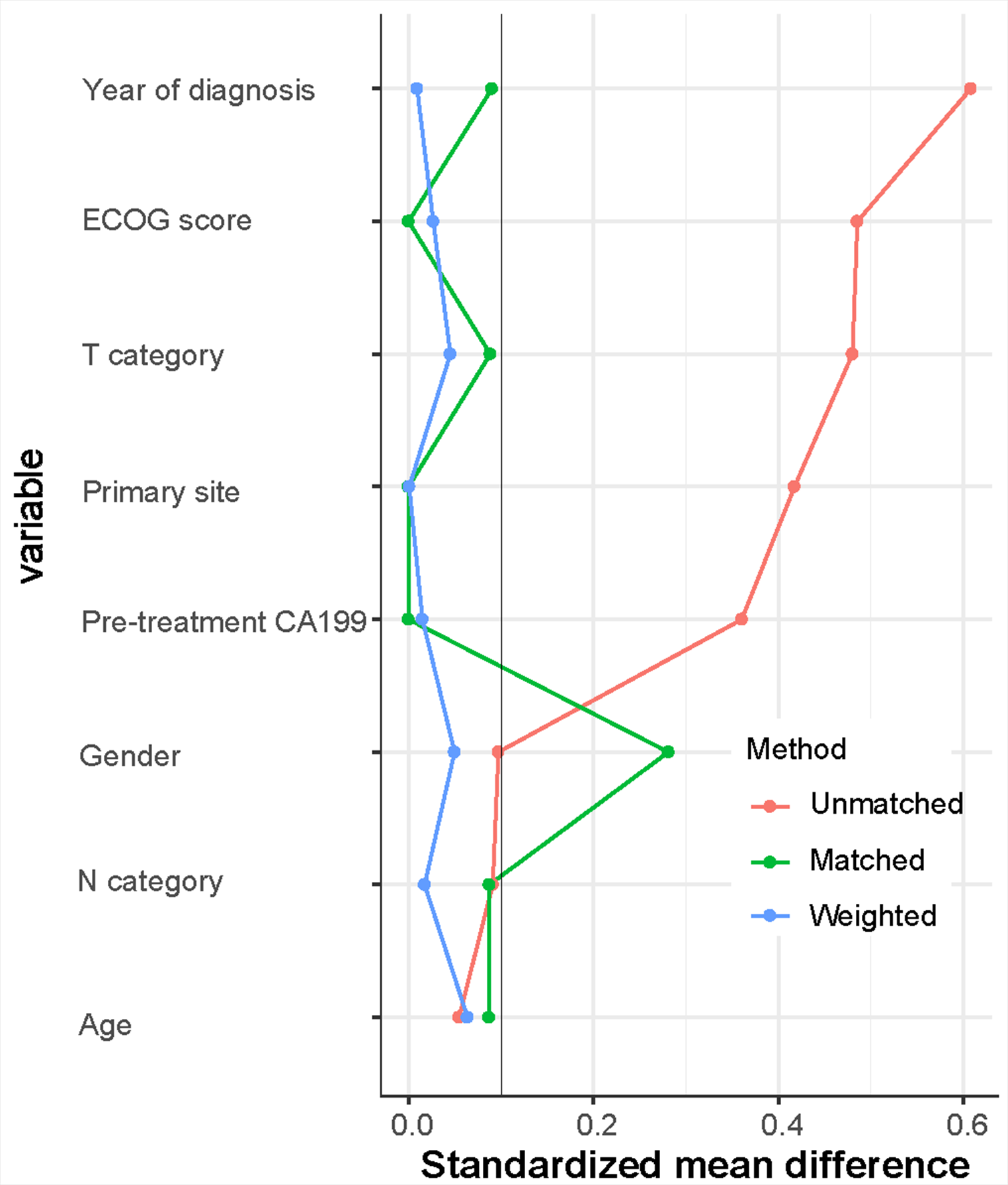

Supplement: Supplementary Figure 1 — Plot of the balance evaluated before and after matching/weighting. [file Image_1.tiff]

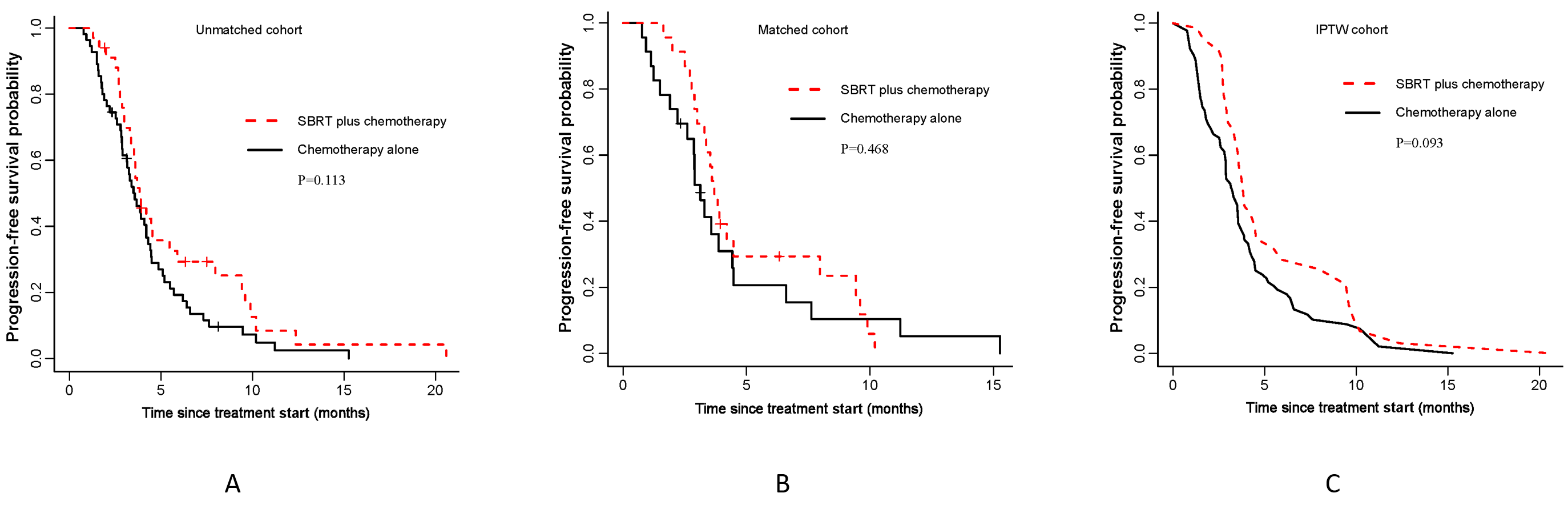

Supplement: Supplementary Figure 2 — Kaplan-Meier curves for PFS. (A) PFS of the unmatched cohort; (B) PFS of the propensity score matched group; (C) PFS of the inverse probability of treatment weight-adjusted group. PFS, progression-free survival; SBRT, stereotactic body radiotherapy. [file Image_2.tif]

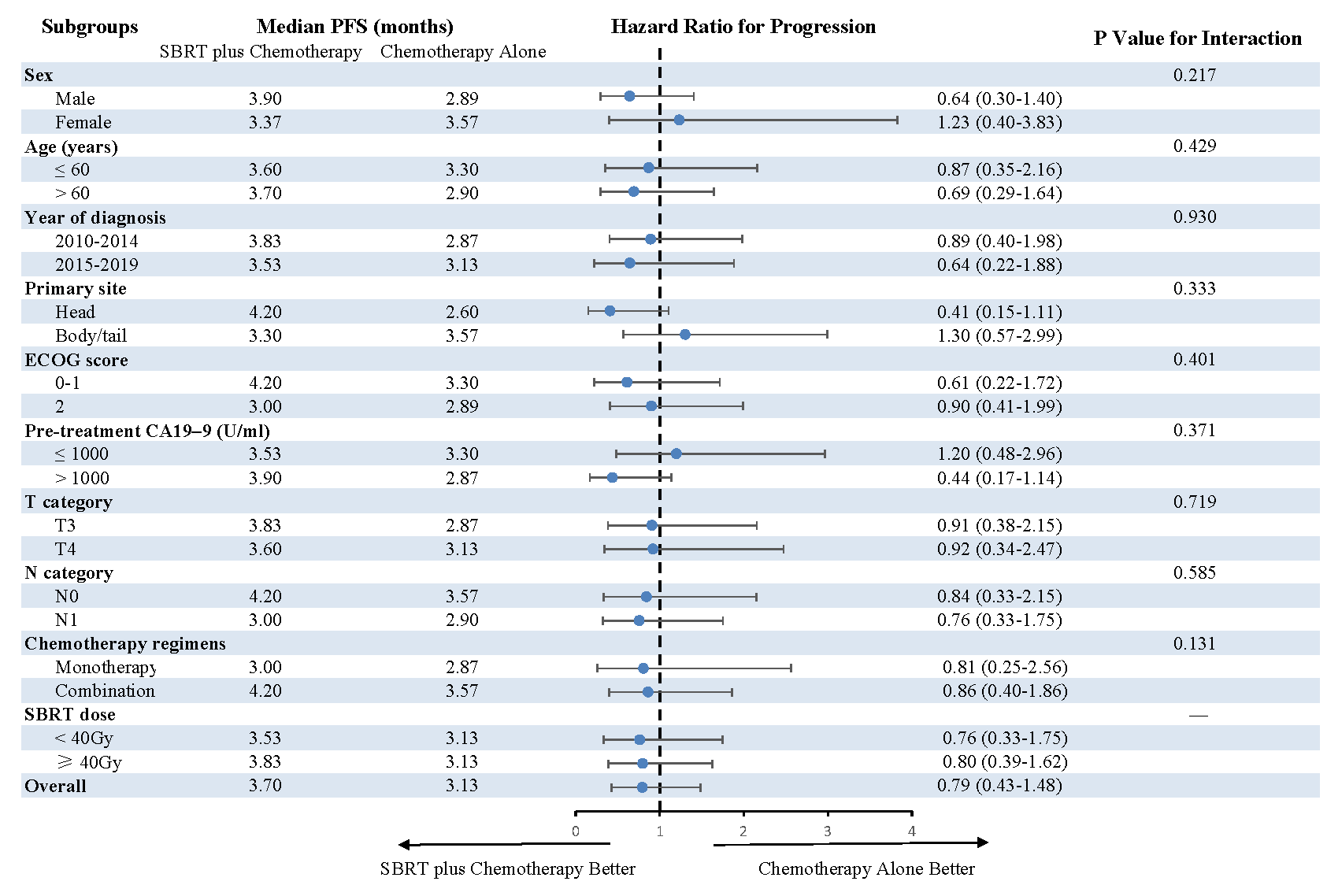

Supplement: Supplementary Figure 3 — Forest plot of PFS subgroup analyses in matched study population. PFS, progression-free survival; SBRT, stereotactic body radiotherapy. [file Image_3.tif]
