# Supplementary material for: Clinical Effects of Stereotactic Body Radiation Therapy Targeting the Primary Tumor of Liver-Only Oligometastatic Pancreatic Cancer
Source: Front Oncol. 2021 May 27;11:659987. doi: 10.3389/fonc.2021.659987 (PMC8190391; doi:10.3389/fonc.2021.659987)
Supplement: Supplementary file 5 [file Table_2.docx]

| **Appendix Table 2**. Comparison of baseline variables between SBRT plus chemotherapy and chemotherapy alone groups in the three dataset with standardized mean difference. | | | | | | | | | |
| --- | --- | --- | --- | --- | --- | --- | --- | --- | --- |
| **Unmatched** | | | | **Propensity Matched** | | | **IPTW** | | |
|  | SBRT plus chemotherapy | Chemotherapy alone | SMD | SBRT plus chemotherapy | Chemotherapy alone | SMD | SBRT plus chemotherapy | Chemotherapy alone | SMD |
| n | 34 | 55 |  | 23 | 23 |  | 26 | 23 |  |
| Gender = Male (%) | 22 (64.7) | 33 (60.0) | 0.097 | 14 (60.9) | 17 (73.9) | 0.281 | 17.7 (67.9) | 16.1 (70.2) | 0.050 |
| Age > 60 (years), (%) | 17 (50.0) | 29 (52.7) | 0.055 | 12 (52.2) | 11 (47.8) | 0.087 | 11.8 (45.3) | 11.1 (48.5) | 0.064 |
| Primary site = Body/tail (%) | 16 (47.1) | 37 (67.3) | 0.417 | 13 (56.5) | 13 (56.5) | <0.001 | 13.5 (51.7) | 11.9 (51.7) | 0.001 |
| ECOG score = 2 (%) | 21 (61.8) | 21 (38.2) | 0.485 | 14 (60.9) | 14 (60.9) | <0.001 | 15.7 (60.4) | 14.2 (61.7) | 0.027 |
| Pre-treatment CA199 > 1000 (U/ml), (%) | 15 (44.1) | 34 (61.8) | 0.360 | 11 (47.8) | 11 (47.8) | <0.001 | 12.2 (47.0) | 10.6 (46.3) | 0.015 |
| T category = T4 (%) | 17 (50.0) | 15 (27.3) | 0.480 | 9 (39.1) | 10 (43.5) | 0.088 | 10.7 (41.2) | 9.0 (39.0) | 0.045 |
| N category = N1 (%) | 17 (50.0) | 30 (54.5) | 0.091 | 12 (52.2) | 13 (56.5) | 0.087 | 14.0 (53.7) | 12.5 (54.6) | 0.017 |
| Year of diagnosis = 2015-2019 (%) | 13 (38.2) | 37 (67.3) | 0.608 | 8 (34.8) | 9 (39.1) | 0.090 | 12.0 (46.3) | 10.5 (45.8) | 0.009 |
| *Abbreviations:* IPTW, inverse probability of treatment weight; CA19-9, carbohydrate antigen 19–9; SBRT, stereotactic body radiotherapy; SMD, standardized mean difference. | | | | | | | | | |
