# Supplementary material for: Clinical Effects of Stereotactic Body Radiation Therapy Targeting the Primary Tumor of Liver-Only Oligometastatic Pancreatic Cancer
Source: Front Oncol. 2021 May 27;11:659987. doi: 10.3389/fonc.2021.659987 (PMC8190391; doi:10.3389/fonc.2021.659987)
Supplement: Supplementary file 6 [file Table_3.docx]

| **Appendix Table 3.** SBRT planning and delivery variables (N = 34 patients) | |
| --- | --- |
| Variables | Median (range) |
| Prescription dose (Gy) | 42.5 (25.0-50.0)/ 5-7 fractions |
| Median BED_10_ (Gy) | 77.7 (37.5-100.0)/ 5-7 fractions |
| Fraction dose (Gy per fraction) | 8 (5.0-10.0) |
| Target size (cm) | 5.8 (4.0-9.0) |
| PTV volumes (cm^3^) | 77.5 (17.8-355.7) |
| PTV coverage (%) | 71.8 (50.6-94.8) |
| Number of beams | 135.5 (71-220) |
| Prescription isodose line (%) | 73 (60-80) |
| Min dose to PTV (Gy) | 20.3 (7.6-40.6) |
| Max dose to PTV (Gy) | 61.2 (35-83.3) |
| HI | 1.37 (1.25-1.67) |
| CI | 1.11 (1-1.35) |
| nCI | 1.57 (1.26-2.32) |
| Pre-SBRT chemotherapy cycles | 1 (0-6) |
| Abbreviations: SBRT, stereotactic body radiotherapy; Gy, gray; BED, biological effective dose; PTV, planning tumor volume; HI, homogeneity index; CI, conformity index; nCI, new conformity index. | |
