# Supplementary material for: Clinical Effects of Stereotactic Body Radiation Therapy Targeting the Primary Tumor of Liver-Only Oligometastatic Pancreatic Cancer
Source: Front Oncol. 2021 May 27;11:659987. doi: 10.3389/fonc.2021.659987 (PMC8190391; doi:10.3389/fonc.2021.659987)
Supplement: Supplementary file 7 [file Table_4.docx]

| **Appendix Table 4** Toxicities in the matched groups (N = 46 patients). | | | | | |
| --- | --- | --- | --- | --- | --- |
| Toxicity | SBRT plus chemotherapy | | Chemotherapy alone | | P |
|  | Grade 1-2 | Grade 3-4 | Grade 1-2 | Grade 3-4 |  |
| **Hematological** |  |  |  |  |  |
| Neutropenia | 7 (30.4%) | 4 (17.4%) | 9 (39.1%) | 6 (26.1%) | 0.484 |
| Anemia | 4 (17.4%) | 3 (13.0%) | 5 (21.7%) | 4 (17.4%) | 0.834 |
| Thrombocytopenia | 3 (13.0%) | 2 (8.7%) | 4 (17.4%) | 3 (13.0%) | 0.802 |
| **Non-hematological** |  |  |  |  |  |
| Hyperbilirubinemia | 1 (4.3%) | 0 (0.0%) | 2 (8.7%) | 0 (0.0%) | 1.000 |
| Elevated ALT | 2 (8.7%) | 0 (0.0%) | 2 (8.7%) | 0 (0.0%) | 1.000 |
| Elevated AST | 2 (8.7%) | 0 (0.0%) | 2 (8.7%) | 0 (0.0%) | 1.000 |
| Fatigue | 10 (43.5%) | 0 (0.0%) | 11 (47.8%) | 0 (0.0%) | 0.767 |
| Anorexia | 8 (34.8%) | 0 (0.0%) | 8 (34.8%) | 0 (0.0%) | 1.000 |
| Diarrhea | 2 (8.7%) | 0 (0.0%) | 1 (4.3%) | 0 (0.0%) | 1.000 |
| Constipation | 1 (4.3%) | 0 (0.0%) | 1 (4.3%) | 0 (0.0%) | 1.000 |
| Nausea | 9 (39.1%) | 0 (0.0%) | 10 (43.5%) | 0 (0.0%) | 0.765 |
| Vomiting | 2 (8.7%) | 0 (0.0%) | 3 (13.0%) | 0 (0.0%) | 1.000 |
| Pain | 1 (4.3%) | 0 (0.0%) | 0 (0.0%) | 0 (0.0%) | 1.000 |
| Nephrotoxic | 2 (8.7%) | 1 (4.3%) | 2 (8.7%) | 1 (4.3%) | 1.000 |
| GI Bleeding | 0 (0.0%) | 1 (4.3%) | 0 (0.0%) | 0 (0.0%) | 1.000 |
| Duodenal ulcer | 0 (0.0%) | 1 (4.3%) | 0 (0.0%) | 0 (0.0%) | 1.000 |
| *Abbreviations:* ALT, alanine transaminase; AST, aspartate aminotransferase; GI, gastrointestinal. | | | | |  |
